# Risk factors for infection with chikungunya and Zika viruses in southern Puerto Rico: A community-based cross-sectional seroprevalence survey

**DOI:** 10.1371/journal.pntd.0010416

**Published:** 2022-06-13

**Authors:** Laura E. Adams, Liliana Sánchez-González, Dania M. Rodriguez, Kyle Ryff, Chelsea Major, Olga Lorenzi, Mark Delorey, Freddy A. Medina, Jorge L. Muñoz-Jordán, Grayson Brown, Marianyoly Ortiz, Stephen H. Waterman, Vanessa Rivera-Amill, Gabriela Paz-Bailey

**Affiliations:** 1 Division of Vector-borne Diseases, Centers for Disease Control and Prevention, San Juan, Puerto Rico; 2 Puerto Rico Vector Control Unit, San Juan, Puerto Rico; 3 Ponce Health Sciences University/Ponce Research Institute, Ponce, Puerto Rico; Université de Montréal, CANADA

## Abstract

Chikungunya virus (CHIKV) caused a large outbreak in Puerto Rico in 2014, followed by a Zika virus (ZIKV) outbreak in 2016. Communities Organized for the Prevention of Arboviruses (COPA) is a cohort study in southern Puerto Rico, initiated in 2018 to measure arboviral disease risk and provide a platform to evaluate interventions. To identify risk factors for infection, we assessed prevalence of previous CHIKV infection and recent ZIKV and DENV infection in a cross-sectional study among COPA participants. Participants aged 1–50 years (y) were recruited from randomly selected households in study clusters. Each participant completed an interview and provided a blood specimen, which was tested by anti-CHIKV IgG ELISA assay and anti-ZIKV and anti-DENV IgM MAC-ELISA assays. We assessed individual, household, and community factors associated with a positive result for CHIKV or ZIKV after adjusting for confounders. During 2018–2019, 4,090 participants were enrolled; 61% were female and median age was 28y (interquartile range [IQR]: 16–41). Among 4,035 participants tested for CHIKV, 1,268 (31.4%) had evidence of previous infection. CHIKV infection prevalence was lower among children 1–10 years old compared to people 11 and older (adjusted odds ratio [aOR] 2.30; 95% CI 1.71–3.08). Lower CHIKV infection prevalence was associated with home screens (aOR 0.51; 95% CI 0.42–0.61) and air conditioning (aOR 0.64; 95% CI 0.54–0.77). CHIKV infection prevalence also varied by study cluster of residence and insurance type. Few participants (16; 0.4%) had evidence of recent DENV infection by IgM. Among 4,035 participants tested for ZIKV, 651 (16%) had evidence of recent infection. Infection prevalence increased with older age, from 7% among 1–10y olds up to 19% among 41–50y olds (aOR 3.23; 95% CI 2.16–4.84). Males had an increased risk of Zika infection prevalence compared with females (aOR 1.31; 95% CI 1.09–1.57). ZIKV infection prevalence also decreased with the presence of home screens (aOR 0.66; 95% CI 0.54–0.82) and air conditioning (aOR 0.69; 95% CI 0.57–0.84). Similar infection patterns were observed for recent ZIKV infection prevalence and previous CHIKV infection prevalence by age, and the presence of screens and air conditioners in the home decreased infection risk from both viruses by as much as 50%.

## Author summary

We assessed baseline seroprevalence and risk factors for previous CHIKV or recent ZIKV infection among 4,035 participants in the Communities Organized to Prevent Arboviruses (COPA) study. Participants were recruited among people 1–50 years old living in 38 study clusters in the Ponce municipality of southern Puerto Rico. Among 4,035 participants, 31% had evidence of previous CHIKV infection, and 16% had evidence of recent ZIKV infection. For both viruses, older age was associated with greater risk for infection; male sex was also associated with higher rates of ZIKV infection. Seroprevalence varied by study cluster, with similar patterns between the two viruses. Home screens and air conditioning in the home were significant protective factors against infection in the multivariate analysis for both CHIKV and ZIKV.

## Introduction

Dengue (DENV), chikungunya (CHIKV), and Zika (ZIKV) viruses are all transmitted by *Aedes* spp mosquitoes and cause acute febrile illness worldwide. Puerto Rico is a tropical island and unincorporated territory of the United States located in the Caribbean Sea, with a population of approximately 3.2 million in 2018. [1] DENV is an endemic flavivirus in Puerto Rico, with circulation of all four dengue viruses. Transmission is seasonal, with the highest number of cases usually reported during July–November, and cyclical outbreaks every 3–5 years since island-wide dengue surveillance began in 1969. [2] DENV seroprevalence in Puerto Rico has been reported to be greater than 90% among adults, and near 50% among children 9–18 years old. [3–5] CHIKV is an alphavirus associated with acute febrile illness and severe polyarthralgias. [6] It was introduced in Puerto Rico in 2014 and caused an explosive outbreak estimated to have infected 25% of the population. [7] ZIKV is a flavivirus and can cause symptoms including rash, fever, and arthralgia, and has also been linked to congenital abnormalities and Guillain-Barre syndrome. [8,9] ZIKV cases were first identified in Puerto Rico in late 2015, followed by an outbreak with over 39,000 confirmed cases peaking in August 2016. [10] The last confirmed Zika virus disease cases were detected in early 2018. [11]

Seroprevalence studies are critical to gather more accurate estimates of infection and population-level immunity in endemic areas. Laboratory-confirmed arboviral disease cases reported through traditional surveillance systems often represent a small proportion of all infections. In addition to asymptomatic cases, people with mild to moderate symptomatic infection might not seek healthcare or have a specimen collected for confirmatory testing. Seroprevalence data can also help inform the need and relevance of vaccines and other prevention programs. For example, models indicate that the dengue vaccine Dengvaxia, which is only recommended for people with previous DENV infection, would be most cost-effective in settings with high dengue seroprevalence. [12,13] Additionally, seroprevalence and seroincidence estimates are needed to inform study designs to evaluate the effectiveness of arboviral disease interventions including vaccines or vector control efforts.

The Communities Organized to Prevent Arboviruses (COPA) project was initiated in southern Puerto Rico in early 2018 to better understand, control, and prevent diseases spread by *Aedes* spp mosquitoes. The project is a community-based cohort study with the goal to better describe arboviral seroprevalence and seroincidence, as well as evaluate arboviral disease prevention strategies. We sought to describe participant characteristics, seroprevalence of previous CHIKV infection, and seroprevalence of recent or acute infection with DENV, CHIKV, and ZIKV in a baseline cross-sectional assessment. Acute infection seroprevalence was defined as detection of viral RNA by RT-PCR, while recent infection was defined as detection of IgM antibodies. [14] Previous infection was defined as the identification of IgG antibodies, which can be detectable for years after infection. [15,16] We also aimed to assess risk factors for previous or recent arbovirus infection at the individual, household, and community level, and correlations with entomologic surveillance data.

## Methods

### Ethics statement

Approval for the COPA project was obtained from the Ponce Medical School Foundation, Inc. Institutional Review Board (protocol number 171110-VR). Written consent to participate was obtained from all adult participants and emancipated minors; parental written consent and participant assent was obtained for children.

### Research setting, study design, and participant selection

The municipality of Ponce, located in southern Puerto Rico, was chosen as the study site due to historically high arboviral disease incidence and existing study infrastructure in the area. [17] Ponce has a tropical climate, with an average annual temperature of 77.8 °F and 32.9 inches of rain per year. [18] A total of 38 study clusters with an estimated population of 73,000 residents were selected from across the municipality, using geocoded historic passive case surveillance data from previous outbreaks of DENV, CHIKV, and ZIKV to identify areas with elevated incidence of arboviral disease. COPA power calculations were designed for the cohort to measure a 50% difference in arboviral incidence over a 3-year intervention period using t-tests in a stratified design. Three-year incidence was assumed to match a historical incidence of about 0.09. The sample size calculations resulted in a goal of 100 participants in each of 38 clusters. A separate sample size computation for the cross-sectional seroprevalence survey was not performed for this analysis.

Within study clusters, all potential household structures were identified using ArcGIS data files and assigned unique identifiers; from this list approximately 300 households were randomly selected for participation using SAS 9.4. Selected households were visited up to three times on different days to offer enrollment, including at least one Saturday. If less than 100 participants per cluster were recruited, a new random selection of households was generated for field teams. Household visit tracking was performed using a house tracking application named HTrack. [19] Study participation was offered to all household members in selected households that met the eligibility criteria, which included people aged 1–50 years old who slept in the house at least 4 nights per week and had no plans to move in the next 6 months. Households in the study area with no residents aged 1–50 years were excluded from the study.

### Questionnaire

All participants were administered a questionnaire capturing information about the household, history of febrile illness, and personal protective behaviors surrounding mosquito avoidance and prevention. Information applicable to all members of the household, including annual household income, water source, presence of screens and air conditioning, were asked to one household representative. Household representatives were selected as an adult 21 years or older or an emancipated minor present at the time of the visit. All participants 7–50 years old were asked about demographics, clinical history, and knowledge, attitudes and practices about mosquito control. For participants under 7 years of age, the knowledge, attitudes, and practices survey was not administered, and the parent or guardian was asked to provide responses to demographic and clinical history questions. Data were collected on tablets with automated checks to reduce missing or incorrect data, and later reviewed by staff to identify potential errors in data entry or data capture. If key information was missing or inconsistent with other responses, participants were contacted by phone to clarify or complete the relevant sections of the questionnaire.

### Laboratory testing

Serum specimens were collected from all participants. Specimens were tested for the presence of anti-CHIKV IgG using the InBios Chikjj Detect IgG ELISA to assess evidence of previous CHIKV infection, which is reported to have a sensitivity of 91% and specificity of 100%. [20] Although length of CHIKV IgG persistence is not well defined, it has been detected at least one year after infection, and was found to provide immune protection against CHIKV infection up to 40 years after exposure. [16,21] Due to concerns about cross-reaction between DENV and ZIKV, IgG testing for the two flaviviruses was not performed in the initial assay. To assess evidence of recent arboviral infection, all specimens were tested with the CDC anti-ZIKV IgM antibody capture enzyme-linked immunosorbent assay (ELISA) [22], the InBios DENV Detect IgM capture ELISA [23], and the CDC anti-CHIKV IgM capture ELISA [24]. Evidence of acute infection was assessed by testing participants who reported a febrile illness in the 7 days before specimen collection by RT-PCR for DENV, CHIKV, and ZIKV using the Trioplex assay. [25]

### Entomological surveillance

Autocidal gravid ovitraps (AGO traps), which have previously been reported to show a correlation between mosquito abundance and arboviral disease risk, were placed for *Aedes aegypti* surveillance in all clusters, with at least one trap per 50 square meters of the study area. [26] AGO traps were serviced weekly by vector control staff, and the number of female *Ae*. *aegypti* captured was recorded per trap. To assess associations between the number of mosquitoes and risk for arboviral disease, we took the average number of female *Ae aegypti* mosquitoes captured per trap by week and by cluster during April 2018–August 2020.

### Statistical analysis

We summarized participant information about demographics, personal protective behaviors, and household-level factors that could affect arboviral infection risk; we also assessed mosquito levels found through entomological surveillance. Bivariate analyses were performed using chi-square tests to assess factors associated with previous infection with CHIKV, defined as a positive result on the CHIKV IgG ELISA, or recent infection with ZIKV, defined as a positive result on the ZIKV IgM ELISA. Statistical significance was defined as P<0.05. Previous infection with CHIKV was used due to the outbreak in 2014, and lack of population-level data to inform infection rates in Puerto Rico. Recent infection with ZIKV was selected as an indicator due to the more recent outbreak of the virus in 2016, and evidence or prolonged persistence of ZIKV IgM. [27] We used generalized linear mixed models to calculate adjusted Odds Ratios (aORs) for risk factors for a positive CHIKV IgG or ZIKV IgM result, using household and study cluster as random effects, and including factors that were significant at the bivariate level. We mapped seroprevalence at the cluster level with mosquito surveillance findings in a bivariate choropleth map to show the potential overlap between high mosquito levels during the study period and previous CHIKV or ZIKV infection. This created nine categories, capturing all options of low, medium, and high seroprevalence and low, medium, and high mosquito counts. The nine categories were selected by splitting each measure (*Aedes aegypti* counts, CHIKV IgG seroprevalence, and ZIKV IgM seroprevalence) into even terciles. We also assessed possible associations between average mosquito counts during the study period and previous arboviral infection using Pearson’s correlation coefficient.

## Results

During April 2018–May 2019, field teams visited 28,213 households in the 38 clusters; 2,566 (9.1%) were recruited into COPA. Additional figures on participant recruitment, exclusions, and the time period described can be found in the supporting information (S1 and S2 Figs and S1 Table). Non-participating households included 10,479 (37.1%) ineligible, 7,586 (26.9%) not contacted after at least 3 attempts, 5,197 (18.4%) vacant or not a home, and 2,385 (8.5%) refusals to participate. In participating households, the response rate among eligible household members was 73.5%. A total of 248 households were discontinued due to a change in cluster boundaries during the study, as well as 37 households with participants greater than 50 years of age due to a change in eligibility criteria to focus on the younger population at higher risk for arboviral disease.

Final participants were recruited from 2,281 households across the 38 clusters, with a median of 51 (range 26–174) participating households per cluster. Among the 2,281 households, 4,090 participants were recruited, with an average of 1.8 participants per household. A total of 4,035 participants had laboratory results available and were included in the analysis. Participant age ranged from 1–50 years; median age was 28 (IQR 16–41) years, and 61% were female. (Table 1). Nearly half of the participants (44.3%) lived in a household with an annual income less than $10,000, and 66.6% of participants were on public insurance (Medicare/Medicaid). When asked about personal and household mosquito protective factors, 44.8% of participants reported they had used insect repellant in the past 30 days, 63.4% had screens on the windows or doors in their home, and 68.7% had air conditioning in the home.

**Table 1:** Chikungunya (CHIKV) IgG and Zika (ZIKV) IgM results by demographic characteristics for COPA participants. Columns show descriptive characteristics of all participants, as well as the percent of each group with positive results for CHIKV IgG or ZIKV IgM.

			Participants positive for CHIKV IgG			Participants positive for ZIKV IgM		
	Total	%	N	%	P-value	N	%	P-value*
**All participants**	**4,035**	**100.0**	**1,268**	**31.4**		**651**	**16.1**	
Female	2,433	60.3	759	31.2	0.70	365	15.0	0.02
Male	1,602	39.7	509	31.8		286	17.9	
Pregnant	36	0.9	10	27.8	0.64	7	19.4	0.59
**Age category in years**								
1 to 10	489	12.1	105	21.5	<0.01	34	7.0	<0.01
11 to 20	895	22.2	295	33.0		157	17.5	
21 to 30	792	19.6	269	34.0		121	15.3	
31 to 40	809	20.1	261	32.3		140	17.3	
41 to 50	1,050	26.0	338	32.2		199	19.0	
**Education level** ^#^								
Grade 0 to 11	1,243	30.8	380	30.6	<0.01	186	15.0	<0.01
Completed grade 12/GED	1,074	26.6	414	38.6		218	20.3	
College or graduate degree	1,648	40.8	450	27.3		240	14.6	
Other/no response	70	1.7	24	34.3		7	10.0	
**Employment type**								
Full time or business owner	873	21.6	239	27.4	<0.01	130	14.9	<0.01
Student, part-time, casual, or informal work	1,927	47.8	571	29.6		291	15.1	
Homemaker, retired, unemployed, or unable to work	1,065	26.4	426	40.0		220	20.7	
Other/no response	170	4.2	32	18.8		10	5.9	
**Insurance type**								
Public	2,686	66.6	953	35.5	<0.01	474	17.7	<0.01
Private	1,066	26.4	223	20.9		135	12.7	
Other, none, no response	283	7.0	92	32.5		42	14.8	
**Annual household income**								
<$10,000	1,787	44.3	674	37.7	<0.01	355	19.9	0.33
$10,000-<20,000	716	17.7	247	34.5		100	14.0	
>=$20,000	1,290	32.0	278	21.6		167	13.0	
No response	242	6.0	69	28.5		29	12.0	
**Repellant use in past 30 days**								
Yes	1,806	44.8	596	33.0	0.05	317	17.6	0.03
No	2,229	55.2	672	30.2		334	15.0	
**Screens in windows and doors**								
Yes	2,559	63.4	637	24.9	<0.01	350	13.7	<0.01
No	1,476	36.6	631	42.8		301	20.4	
**Air conditioning in home**								
Yes	2,773	68.7	762	27.5	<0.01	383	13.8	<0.01
No	1,262	31.3	506	40.1		268	21.2	
**Chikungunya IgG**								
Positive	1,268	31.4				295	23.3	<0.01
Negative	2,767	68.6				356	12.9	

*Chi-square tests used to assess significance (P<0.05) for categorical variables.

^#^Grades correspond to U.S. grade levels. The category “Grade 0 to 11” includes children starting education at 5–6 years old, and represents an education level of less than high school graduation, which is usually completed when students are 17–18 years old. “Completed grade 12/GED” represents completion of high school or an equivalent level of education. General Educational Development (GED) tests are a group tests that provide evidence that the test taker has high school-level academic skills, and is equivalent to a high-school diploma.

### Laboratory testing

Testable specimens were available for 4,035 (98.7%) of the 4,090 participants; hemolyzed specimens were not tested. Among specimens tested, 1,268 (31.4%; 95% Confidence Interval 30.0%–32.9%) participants were positive for CHIKV IgG (Table 1). Serologic testing identified 651 (16.1%; 95% CI 15.0%–17.3%) positive for anti-ZIKV IgM antibodies, 16 (0.4%; 95% CI 0.2%–0.6%) participants positive for anti-DENV IgM antibodies, and 28 (0.7%; 95% CI 0.4%–1.0%) positive for anti-CHIKV IgM antibodies. Among 216 participants reporting fever in the 7 days before interview, all were negative by RT-PCR for DENV, ZIKV, and CHIKV. A total of 319 participants had positive results from serologic testing for two or more arboviruses; this included 7 participants with positive results for CHIKV IgG, ZIKV IgM, and DENV IgM, and 3 participants with positive results for CHIKV IgG, ZIKV IgM, and CHIKV IgM. The remaining 309 participants with more than one positive result included 285 participants with positive results by CHIKV IgG and ZIKV IgM, 18 participants with positive results by CHIKV IgG and CHIKV IgM, 5 participants with positive results by ZIKV IgM and DENV IgM, and 1 participant with positive results for ZIKV IgM and CHIKV IgM.

### Entomologic surveillance

**Fig 1:**
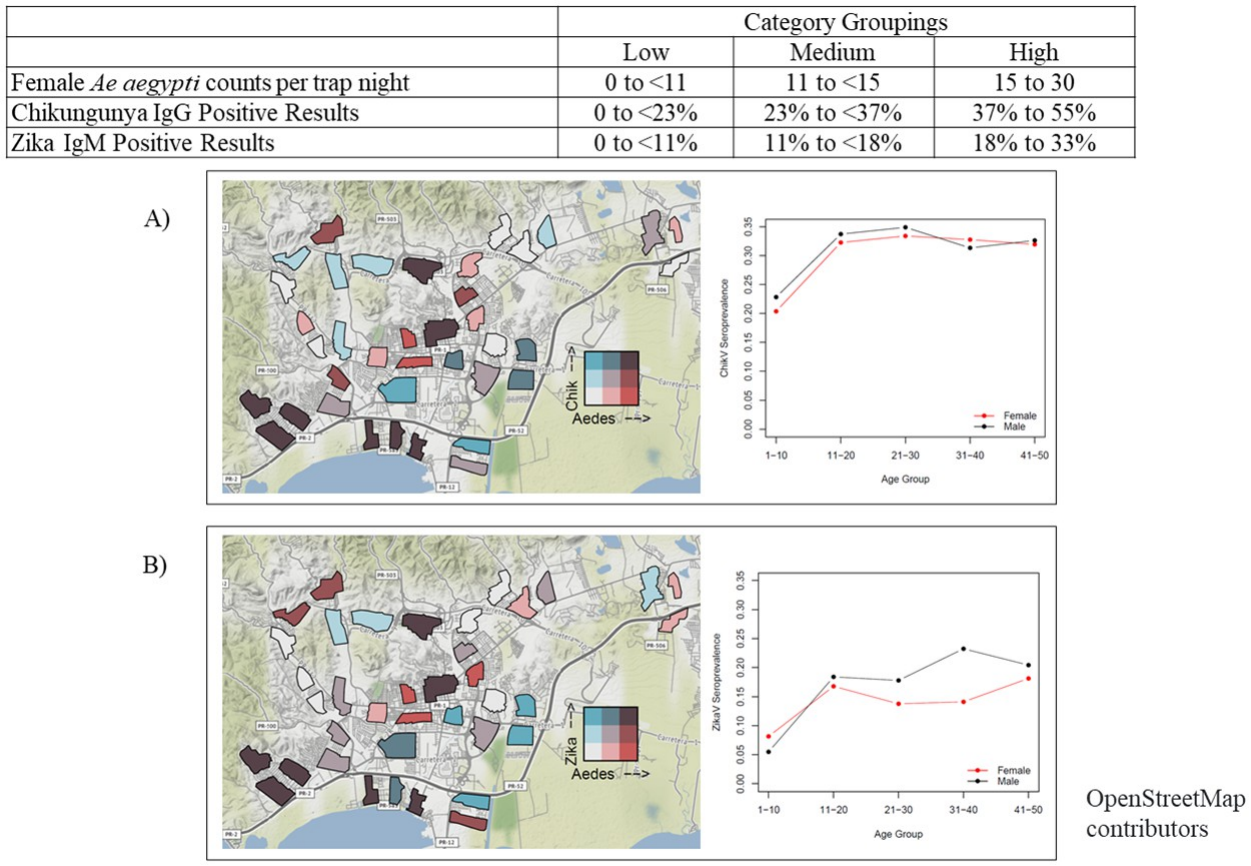
Chikungunya virus (A) and Zika virus (B) seroprevalence by age and sex with *Ae*. *aegypti* mosquito density by cluster. The map base layer was created using data from OpenStreetMap contributors, which is available under the Open Database License (https://www.openstreetmap.org/copyright).

A total of 727 AGO traps were placed in the 38 clusters, with placement beginning in April 2018 and continuing through August 2020. As traps were placed throughout the recruitment period, time under entomologic surveillance varied for the 38 clusters; mosquito counts were included from the entire surveillance period (April 2018–August 2020). Across all clusters, a mean of 7.1 (95% CI 7.0–7.2) female *Ae*. *aegypti* mosquitoes were captured weekly per trap. The average mosquito counts per cluster overlaid with seroprevalence data by cluster for CHIKV IgG and ZIKV IgM (Fig 1).

### Factors associated with infection

In the bivariate analysis, previous CHIKV infection was associated with multiple factors, including older age, lower education level, employment type, public insurance, lower household income, study cluster, insect repellant use, and the absence of screens and air conditioning in the home. No significant differences were observed by reported mosquito control practices including regular elimination of standing water, covering water-holding containers, cleaning trash and debris around the home, use of insecticide indoors or outdoors, or burning mosquito-repellant candles or coils. In the multivariate analysis, risk factors that remained significant included age older than 10 years (OR 2.30; 95% CI 1.71–3.08), public insurance (OR 1.49; 95% CI 1.20–1.84), and lower education (Table 2). Protective factors against previous CHIKV infection included the presence of screens in doors and windows (OR 0.51; 95% CI 0.42–0.61) and air conditioning (OR 0.64; OR 0.54–0.77) in the home.

**Table 2:** Risk factors for previous chikungunya virus infection (IgG) or recent Zika virus infection (IgM) from the multivariate analysis.

Participant or household characteristic	CHIKV IgG			ZIKV IgM		
	aOR*	95% CI		aOR*	95% CI	
**Male Sex**				**1.31**	**1.09**	**1.57**
**Age category in years**						
1 to 10	Ref			Ref		
11 to 50	**2.3** **0**	**1.71**	**3.08**	**3.23**	**2.16**	**4.84**
**Education level**						
Grade 0–11	1.10	0.89	1.36	1.11	0.86	1.42
Grade 12/GED	**1.28**	**1.06**	**1.56**	1.20	0.96	1.50
College or higher education	Ref					
**Insurance**						
Public	**1.49**	**1.20**	**1.84**	1.24	0.98	1.58
Private	Ref			Ref		
**Screens in windows and doors**	**0.51**	**0.42**	**0.61**	**0.66**	**0.54**	**0.82**
**Air conditioning in home**	**0.64**	**0.54**	**0.77**	**0.69**	**0.57**	**0.84**

*Adjusted Odds Ratio (aOR)

For ZIKV, male sex, older age, public insurance, study cluster, repellant use, and lack of screens and air conditioning in the home were associated with higher ZIKV IgM levels in the bivariate analyses. In the multivariate model, male sex (OR 1.31; 95% CI 1.09–1.57) and age older than 10 years (OR 3.23; 95% CI 2.16–4.84) were associated with increased risk for ZIKV IgM seroprevalence. The presence of screens (OR 0.66; 95% CI 0.54–0.82) and air conditioning (OR 0.69; 95% CI 0.57–0.84) in the home were also significant protective factors in the final model.

Mapping CHIKV IgG seroprevalence in the 38 clusters and *Aedes aegypti* surveillance data identified a moderate correlation (0.5; 95% CI 0.22–0.71) between *Ae*. *aegypti* counts and previous CHIKV infection, with 8 (21.1%) clusters with both high CHIKV seroprevalence and high *Ae*. *aegypti* numbers and 6 (15.8%) clusters with both low CHIKV seroprevalence and low *Ae*. *aegypti* numbers (Fig 1A). For ZIKV IgM, results found a weak correlation between ZIKV IgM seroprevalence and *Ae*. *aegypti* counts (0.39; 95% CI 0.08–0.63); 7 (18.4%) of clusters had high ZIKV IgM seroprevalence and high *Ae*. *aegypti* counts, and 6 (15.8%) had both lower ZIKV IgM seroprevalence and low *Ae*. *aegypti* counts (Fig 1B). Seven of the clusters with high ZIKV IgM seroprevalence and high *Ae*. *aegypti* counts also had high CHIKV seroprevalence.

## Discussion

During 2018–2019, we recruited more than 4,000 participants into the COPA study in southern Puerto Rico and found evidence of previous CHIKV infection in approximately one third of the study population in the cross-sectional survey. Recent ZIKV infection, as evidenced by ZIKV IgM, was identified in one of every six participants—including 7 pregnant women—although recruitment and testing occurred two years after the peak of the ZIKV outbreak in Puerto Rico. Older age was associated with higher seroprevalence of both ZIKV IgM and CHIKV IgG; lower education and public insurance were associated with increased risk for CHIKV IgG only, and male sex was associated for increased risk for ZIKV IgM only. Screens and air conditioning in the home were associated with lower risk for both CHIKV IgG positive results and ZIKV IgM. *Ae*. *aegypti* mosquito counts by cluster showed a moderate association with previous CHIKV IgG seroprevalence, but a weaker association with ZIKV IgM seroprevalence. There was also an association between previous CHIKV infection and ZIKV infection, indicating that the risk factors for arboviral infection remained similar over the two-year period between the two outbreaks in 2014 and 2016. We identified few participants with evidence of recent DENV infection and did not identify any participants with acute infection of DENV, CHIKV, or ZIKV by RT-PCR.

The high level of ZIKV IgM seroprevalence among participants was an unexpected finding. IgM antibodies are usually detectable for at least 12 weeks post-infection; however, ZIKV IgM persistence was reported to be detectable in 73% of participants in one study 12–19 months after illness onset. [27] Although the exact time of infection is unknown for our participants, the large ZIKV outbreak in Puerto Rico—including the municipality containing COPA clusters—occurred during 2016, while COPA specimens were collected in 2018–2019. Our data suggest that ZIKV IgM may remain detectable from infections that occurred two to three years earlier. This also could have affected seroprevalence over the data collection period; however, an analysis of ZIKV IgM seroprevalence by month of sample collection did not identify a significant correlation between time and ZIKV IgM seroprevalence findings. Modeling studies using blood donor data during the ZIKV epidemic estimated a cumulative incidence of 12.9%, which is slightly lower than the finding among COPA participants of 16.1% ZIKV IgM seroprevalence. [28] The overall CHIKV seroprevalence at 31% falls between estimates from blood donor screening in Puerto Rico at 25% and findings from other community-based studies performed in southern Puerto Rico at 45%. [7,29]

Several differences in seroprevalence were observed for both viruses by age and sex. For CHIKV, seroprevalence among participants 11 years and older was similar at ~32%; the lower seroprevalence (21%) among children 1–10 years old is at least in part due to low CHIKV circulation after the large outbreak in 2014, as children born in the years after the outbreak may have had lower risk for infection. ZIKV IgM seroprevalence was a similarly low level among children 1–10 years (7%) compared to 17% among adults. Although children under two years old could have been born after the ZIKV outbreak in 2016, the lower rates of ZIKV were also observed at each age up to 10 years. These findings suggest both an age effect and a period effect, and that children may have been at lower risk for ZIKV than older adults, or that the length of ZIKV IgM persistence could differ by age. Unlike CHIKV IgG, significant differences were also observed by sex, with higher ZIKV IgM seroprevalence among males at all ages over 10 years. This finding contradicts many other ZIKV reports, including surveillance findings in Puerto Rico, which found higher numbers of ZIKV cases among females compared with males [10,30–32]. Our findings suggest higher rates of ZIKV infection in males despite increased case reports among females or possibly differences in ZIKV IgM persistence by sex. Household-based cluster investigations in Puerto Rico in 2016 found similar ZIKV infection rates by sex, but an increased risk for symptomatic infection among females compared to males. Additional studies are needed to better elucidate ZIKV immune responses over time and differences by sex.

Screens and air conditioners are known to be protective against DENV infection [33,34], and the findings here from CHIKV and ZIKV help support this premise for other *Aedes*-transmitted diseases. Indicators of socioeconomic status (SES), including education level, employment type, annual household income, and insurance type were significant in the bivariate analyses for both CHIKV IgG and ZIKV IgM; however, in the multivariate model, insurance type (public) had the strongest association with risk for previous CHIKV infection, and may be the best proxy for SES in our population. Lower SES has previously been associated with DENV infection. [35–37] Although no virologically confirmed acute DENV infections were detected in this study period and less than 1% of participants had positive results by DENV IgM, sampling occurred in a time with low levels of DENV across Puerto Rico, following a cyclical pattern observed for several decades. [2] However, large DENV outbreaks in the Americas and Caribbean region during 2019–2020 [38] increase the risk for future DENV in Puerto Rico.

There are several limitations associated with this study. The outcomes assessed correspond to time periods before the study began, with the CHIKV outbreak in 2014 and the ZIKV outbreak in 2016. Although ZIKV IgM findings were higher than expected, they are likely an underestimate of all previous ZIKV infections due to waning IgM levels over time. Findings in 2018 might not be representative of the risk factors at the time of exposure, both in terms of mosquito density and participant characteristics. In particular, the lack of strong correlations between mosquito counts and previous arboviral infection could be attributable to changes in mosquito populations over time due to the local environment or climate. Findings from the AGO traps might not be representative of all areas of the study clusters; however, previous a previous study with similar AGO surveillance methods found correlations between CHIKV infection risk and entomologic surveillance data from AGO traps. [26] Other risk factors including household and personal protective factors or population mobility could also explain differences in risk not directly related to mosquito populations. However, the strong correlation between positive results for CHIKV and ZIKV indicates that exposures and participant characteristics are likely similar for most participants over the multi-year period. Participants might not be representative of the larger community, as only 9% of households visited were recruited and more than one-third of households visited were ineligible due to resident age greater than 50 years. COPA participants included a slightly higher proportion of females, and a slightly lower proportion of young children, compared to population estimates of the surrounding areas. (S1 Table) The sample could have been biased by selection of people more likely to be home during the day, which could have different risks for arboviral infection than people at work or at school during the day. To minimize this bias, all homes were visited on evenings and weekends to offer opportunities for recruitment to people away during the day. There was also a large proportion of vacant homes, although this was lower that the percent of vacant homes reported in the 2018 American Community Survey (18% vs 24%). [39] Overall, while COPA participants might have slight differences from the underlying population, general findings are likely applicable to the region and potentially other parts of Puerto Rico. Key strengths of the study include a large sample size (>4,000 participants) that includes laboratory results, entomologic data, and interview findings from a population at risk for arboviral infection.

## Conclusion

In the first year of the COPA project in Puerto Rico, different infection patterns were observed among participants for recent ZIKV infection and previous CHIKV infection by age and socioeconomic status. However, the presence of screens and air conditioners in the home decreased infection risk from CHIKV and ZIKV; use of screens and air conditioning should be encouraged in areas with diseases transmitted by *Aedes aegypti* for arboviral disease prevention. These findings provide historical information about recent outbreaks in the island, and a baseline for prevention and control activities in the future.

## Supporting information

S1 Fig Communities Organized to Prevent Arboviruses (COPA) household and participant recruitment. This shows a flowchart detailing participant recruitment and exclusion criteria, both at the household and individual level, for selection of the 4,035 participants with results described in the manuscript.
(TIF)

S2 Fig Communities Organized to Prevent Arboviruses (COPA) year 1 participant enrollment and entomologic surveillance, 2018–2020. This figure shows the timeline for participant and the longer, overlapping period for entomologic surveillance included in the analysis.
(TIF)

S1 Table Age and sex of Communities Organized to Prevent Arboviruses (COPA) participants and residents of Ponce, Puerto Rico. Population estimates for the municipality of Ponce, Puerto Rico were taken from the American Community Survey (ACS) 2018 estimates. COPA participation was limited to people 1–50 years old, and comparisons of percentages by age only included eligible age groups from the ACS (0 to 54 years old); 47,358 people aged 55 and older were excluded from age calculations. All ages were included from the ACS for the comparison by sex.
(XLSX)
