# Supplementary figures and images for: Risk factors for infection with chikungunya and Zika viruses in southern Puerto Rico: A community-based cross-sectional seroprevalence survey
Source: PLoS Negl Trop Dis. 2022 Jun 13;16(6):e0010416. doi: 10.1371/journal.pntd.0010416 (PMC9191703; doi:10.1371/journal.pntd.0010416)

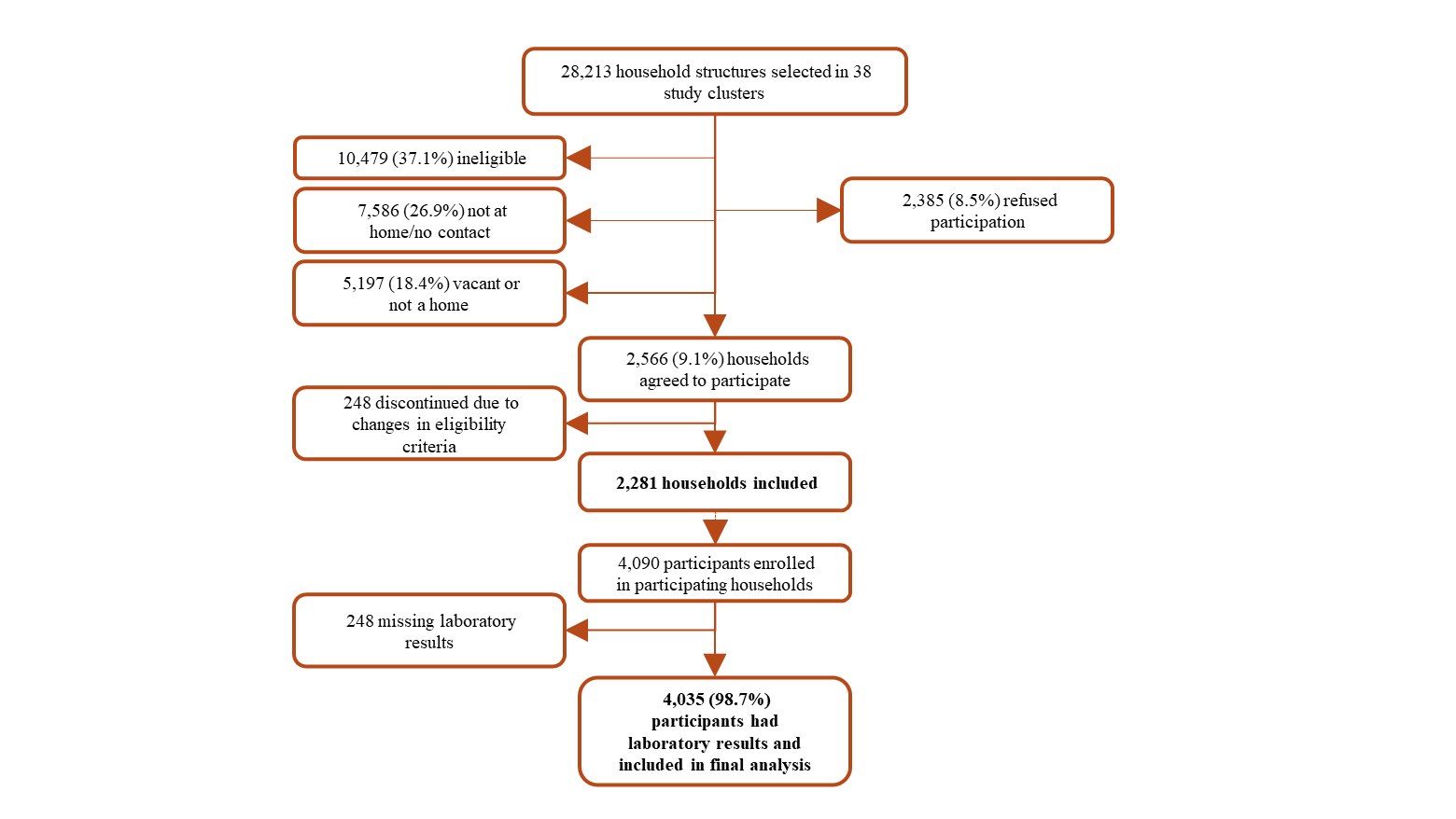

Supplement: S1 Fig — This shows a flowchart detailing participant recruitment and exclusion criteria, both at the household and individual level, for selection of the 4,035 participants with results described in the manuscript. (TIF) [file pntd.0010416.s001.tif]

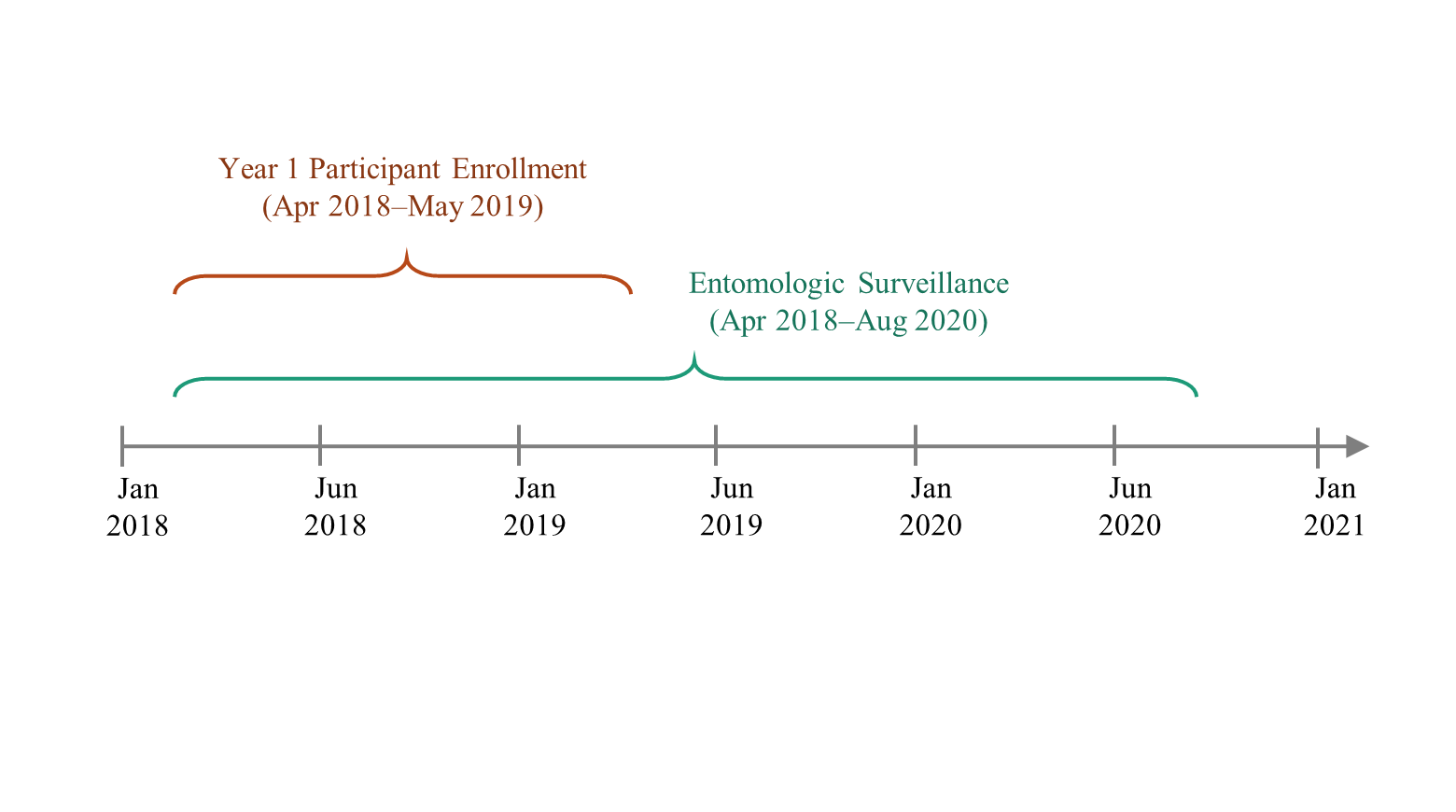

Supplement: S2 Fig — This figure shows the timeline for participant and the longer, overlapping period for entomologic surveillance included in the analysis. (TIF) [file pntd.0010416.s002.tif]
